# Evaluation of the Shear Bond Strength of Nanocomposite on Carious and Sound Deciduous Dentin

**DOI:** 10.5005/jp-journals-10005-1129

**Published:** 2012-02-24

**Authors:** Seema Deshmukh, B Nandlal

**Affiliations:** Senior Lecturer, Department of Pediatric and Preventive Dentistry JSS Dental College and Hospital, JSS University, Mysore, Karnataka India, e-mail: dr_seemadeshmukh07@yahoo.co.in; Professor, Department of Pediatric and Preventive Dentistry, JSS Dental College and Hospital, JSS University, Mysore, Karnataka, India

**Keywords:** Carious dentin, Sound dentin, Conventional composite, Nanocomposite

## Abstract

**Aim:** The aim of the study was to evaluate and compare the shear bond strength of conventional composites with nanocomposites in carious and sound deciduous dentin with the use of self-etching adhesive.

**Methodology:** Human primary molars were ground to obtain flat dentin surfaces and divided into two groups: Carious dentin and sound dentin group. The carious teeth specimens were prepared by removing infected dentin and area with affected dentin was used for bonding composite. Teeth with carious and sound dentin were subdivided in two groups (n = 15) based on the type of the composite into conventional composite group and nanocomposite group. The composite was bonded to the teeth with self-etching adhesive. All the bonded specimens were stored in distilled water for 24 hours at 37°C before shear bond testing. Independent t-test and analysis of variance were applied to the results.

**Results:** The results indicated that the nanocomposite offered significantly higher bond strength compared to conventional composite. In addition presence of affected dentin significantly reduced the bond strength of both the composite types.

**How to cite this article:** Deshmukh S, Nandlal B. Evaluation of the Shear Bond Strength of Nanocomposite on Carious and Sound Deciduous Dentin. Int J Clin Pediatr Dent 2012;5(1): 25-28.

## INTRODUCTION

Pediatric dental practice requires a restorative material that can be quickly and easily placed with a reliable adhesion to tooth structure. A dislodged filling is an inconvenience to both patient and the dentist. The present day composite has become popular restorative materials for primary anterior and posterior teeth. Another reason for the increased use of composites in pediatric dentistry is the growing demand from parents to provide esthetic restorations for their children.^1^

In recent years, many efforts have been undertaken to develop restorative composite having physicochemical properties similar to those of the natural tooth structure. Improvements of currently used commercial dental restorative composite resins are focused on the reduction of the polymerization shrinkage as well as improvement of mechanical properties, wear resistance, biocompatibility and processing properties.^2^

Dentistry is undergoing yet another change by providing mankind with nanodentistry which will make possible the maintenance of comprehensive oral health by employing nanomaterials. One such change is evident in composites too with the incorporation of nanofillers. Exemplifying a significant advance in the ability of composite resins to provide a clinical benefit traditionally evident in porcelain, a newer material is introduced which consists of organically modified filler particles. The nanofiller used in these composites include alumina silicate powder having a mean particle size of 80 nm. These have superior hardness, strength and excellent handling properties. Development of nanocomposite resin using advanced methacrylate resin has esthetic properties required for anterior restorations and mechanical properties for posterior restoration. Nanofillers are very different from traditional fillers. Due to the presence of specially developed rheologically modified nanofiller particles, a comprehensive resin system has evolved which fulfills the requirements of esthetics as well as function.^3^

Most laboratory bonding studies are done on sound, flat, polished and freshly-cut dentin. Although such results are of great value for the comparative purposes, sound, normal dentin is not the substrate most frequently encountered in clinical situations. Instead, clinicians usually deal with caries affected dentin or sclerotic dentin. Caries affected dentin is partially demineralized, and carious intertubular dentin exhibits a higher degree of porosity than sound intertubular dentin due to mineral loss which can affect the bonding of the restorative material to the tooth. Unfortunately, little work has been done in this regard to assess the resin bonding to caries affected and sclerotic dentin.^4,5^ Therefore, the purpose of this study is to evaluate and compare the shear bond strength of conventional composite and nanocomposite in carious and sound deciduous dentin.

The null hypothesis proposed was that the size of the filler particles incorporated in composite would not affect the bond strength of the restorative material. The objectives of the study were:

1. To evaluate and compare the shear bond strength of conventional composite and nanocomposite.
2. To evaluate the effect of presence or absence of affected dentin on the shear bond strength of restorative material.

## MATERIALS AND METHODS

### Materials used in the Study

Composite resin cements and the self-etching adhesive were used in the study. The self-etching adhesive used was a sixth generation two bottle adhesive. The composite resins used were microhybrid composite and nanocomposite.

### Specimen Preparation

Sixty carious and noncarious primary molars, extracted for various clinical reasons were used. None of the teeth were root filled. It was made sure that the teeth had minimum of one-third of their roots left for the proper anchorage in the acrylic block. The teeth collected were those extracted just 3 months before the study. After extraction the teeth were frozen in physiologic saline. The teeth were disinfected with 0.5% chloramine prior to use. The teeth were sectioned longitudinally buccolingually using a diamond disk. The sectioning was done under continuous supply of saline dropping on the diamond disk. This was done to prevent generation of excess heat during tooth preparation. The carious teeth specimens were prepared such that the selected specimen had the affected dentin on the area where the bonding was to be performed. The affected dentin was conformed by the use of caries detector dye. The teeth were then mounted in the acrylic block. The region to be bonded was then finished with 600 grit silicon carbide paper under running water. This was done to create a standardized smear layer. The teeth were then divided in two groups (Table 1).

**Table 1:** **Table 1: **Mean shear bond strength values (MPa)

*Groups*		*n*		*Conventional composite mean (SD)*		*Nanocomposite mean (SD)*	
Sound dentin		30		11.67 ± 0.43		12.56 ± 0.45	
Carious dentin		30		6.79 ± 0.41		7.59 ± 0.34	

The area above the pulp chamber with dentinal tubules parallel to the long axis of tooth was selected as the area for bonding composite because this area was shown to give consistency in the results. The surface area for adhesion was delimited using an adhesive tape on which holes of 2 mm diameter were made using a rubber dam punch. This was necessary to ensure that the restorative material was inserted into a defined, secure surface area no larger than the one to be tested. For the purpose of stabilizing the tooth while bonding the composite, a bonding jig was prepared with a split Teflon mould of dimensions 3 × 2 mm in which the composite was packed. The Teflon was stabilized with two movable rods, which could be aligned and tightened in position with nuts. The acrylic blocks with the sectioned teeth were stabilized and secured in the bonding jig exposing the bonding site. One drop of liquid each from bottle A and bottle B of self-etching adhesive were mixed in the mixing well and was applied to the dentin for 20s, air dried and light cured for 20s. The Teflon mould was then filled in incremental technique with composite and light cured for 40s. For maintaining the consistency in the study, conventional composite and nanocomposite used belonged to the same manufacturer. All the bonded specimens were left undisturbed for 30 minutes and then stored in distilled water for 24 hours at 37°C before shear bond strength testing. The shear bond testing was done with universal testing machine at a crosshead speed of 1mm/minute using blade parallel to interface between composite and dentin. The fracture site was examined type of failure evaluated (Table 3). The values obtained were calculated in MPa according to area of adhesion and subjected to statistical analysis.

### Statistical Analysis

All the statistical calculations were performed using SPSS software for Windows version 16.0 (Statistical presentation system software, SPSS Inc, New York). The significance levels were fixed at 0.05 (significant), 0.01 (significant), 0.001 (highly significant) levels. Any probability value above 0.05 was considered nonsignificant. In analyzing the results of experiment under methods considered in this research work, the statistical technique employed after estimation of arithmetic and standard deviation were:

1. Independent samples t-test
2. Analysis of variance.

## RESULTS

Mean and standard deviation values are summarized in the Table 1. The results indicated that the mean shear bond strength along with standard deviation values for conventional composite to sound and carious deciduous dentin were 11.67 ± 0.43 MPa and 6.79 ± 0.41MPa respectively and that for nanocomposite bonded to sound and carious dentin were 12.56 ± 0.45 MPa and 7.59 ± 0.34 MPa respectively (Table 1).

One way ANOVA revealed a significant difference in the mean shear bond strength values of different groups (p < 0.001) (Table 2). Nanocomposite group had statistically higher bond strength compared to conventional composites in both carious and sound dentin. Presence of carious dentin significantly reduced the bond strength. Highest bond strength was obtained with nanocomposites bonded to sound dentin.

## DISCUSSION

The majority of the resin composites available now are universal hybrid composites or microhybrid composites. In many clinical studies, several hybrid resin composites showed excellent clinical performance. However, there are controversial findings regarding the performance of the hybrid composites. And also increasing demand for esthetic dental restorations in pediatric dentistry has led to the development of many systems designed to provide sufficient strength and bonding to the tooth structure.^6^

**Table 2:** **Table 2: **One-way ANOVA

*Source of variance*		*Sum of squares*		*df*		*Mean square*		*F*		*p-value*	
Between groups		374.86		3		124.95		746.78		<0.001**	
Within groups		9.37		56		16					
Total		384.23		59						

**Highly significant; df: Degrees of freedom; F: Fisher’s value; p: Probability

**Table 3:** **Table 3: **Percentage-wise distribution of the failure mode at fracture site

*Type of dentin substrate*		*Type of composite*		*Adhesive*		*Cohesive*		*Mixed*	
Sound dentin		Conventional composite		33.3%		33.3%		33.3%	
		Nanocomposite		26.7%		33.3%		40%	
Carious dentin		Conventional composite		46.7%		13.3%		40%	
		Nanocomposite		53.3%		26.7%		20%	

Recently, a new category of resin composites were developed and named nanofilled composites. Restorative composites systems made by the use of nanotechnology can offer high translucency, high polish retention similar to that of microfilled composites while maintaining physical properties and wear equivalent to several hybrid composites.^6^Nanofillers incorporated in the nanocomposites are different from traditional composites. They are manufactured synthetically by chemical processes and are used to produce building blocks on molecular scale. The nanomeric particles are monodisperse nonaggregated and nonagglomerated silica nanoparticles having esthetic properties required for anterior restoration and mechanical properties required for posterior restorations.^7,16^

However, most of the studies with these newly introduced materials are done on normal dentin for convenience. Clinically, most bonding substrates are not normal dentin but rather caries affected dentin and sclerotic dentin. The mechanism of resin adhesion to caries dentin is different from that of adhesion to normal dentin, which may affect the bond strength. There are various factors that can have affect on the bond strength to the carious dentin. Hence, it is essential to simulate the clinical situation to know the properties of the restorative material.^4,8^

Various areas on tooth have been selected in different studies for bonding the composites to tooth. Consistency in the results of the bond strength was obtained when the area selected was such that the dentinal tubules parallel to the long axis of teeth. The reason for this is increase in intertubular dentin area which is less mineralized and contains more collagen fibers which is favorable for newer generation bonding agents.^9-11^

In order to exclude the possible influences of different bonding systems on bond strength, two different composite resins were used with similar bonding system. Self-etch adhesive systems is preferred as there is a trend to move away from older multi component bonding systems towards more simplified, consolidated adhesives that are more user friendly. With self-etch approach, there is no etching, rinsing and drying steps, which reduces not only application time but also sensitivity of technique and risk manual errors during application procedures.^12-14^

The results of the present study indicated that higher mean shear bond strength was obtained with nanocomposite in both sound (12.56 ± 0.45 MPa) and carious dentin (7.59 ± 0.34 MPa), when compared to conventional composite which showed 11.67 ± 0.43 MPa for sound and 6.79 ± 0.41 MPa for carious dentin. The reason for the higher strength with nanocomposite probably might be due to the altered structure of the nanocomposite with nanomeric filler particles. Incorporation of these nanomeric filler particles increases the filler loading and thereby improving the mechanical and physical properties of the material. Similar results were obtained by Sumita B Mitra and Brian N Holmes, where in the mechanical properties of nanocomposite are found to higher than those of the conventional composites. The possible reason for this could be that nanofiller particles are fundamentally different from filler particles in conventional composites. The primary particles in conventional composites typically aggregate in fibrous, low density structures. This fibrous structure of the conventional composites limits filler loading and results in poor handling. In nanocomposites the filler loading is more uniform. The filler particles in nanocomposites are more compactly placed and also thus allow more amount of resin to be incorporated. Thus, the mechanical properties obtained with nanocomposites are found to be higher than those of conventional composites.^3^

The findings of this study showed that shear bond strength obtained for sound dentin significantly was higher than that obtained with carious dentin with both conventional and nanocomposite. The values obtained with the carious dentin were 6.79 ± 0.41MPa for conventional dentin and 7.59 ± 0.34 MPa for nanocomposite which were significantly lower than that obtained for sound dentin (11.67 ± 0.43 MPa with conventional composites and 12.56 ± 0.45 MPa with nanocomposite). Similar results were obtained by Alp Erdin Koyuturk et al, M Yoshima et al and M Yoshima, FR Tay et al where in the caries affected dentin showed less bond strength when compared to sound dentin under similar conditions.^4,17,18^

The probable reasons for reduced bond strength with carious dentin are:

1. Carious dentin is softer than sound dentin because it is partially demineralized.
2. Intertubular dentin which is a very essential area for efficient bonding exhibits higher degree of porosity in the carious dentin when compared to sound dentin.
3. Presence if acid resistant mineral casts within the dentinal tubules due to the repeated remineralization and demineralization. These areas hamper the resin infiltration in the dentinal tubules and hence proper hybrid layer is not formed.
4. Further the structure of the carious dentin is also different from sound dentin that is the mineral occupying the interfibrillar space is different from normal apatite which might influence the hybrid layer formation and chemical interactions with carboxylic and phosphate derivatives of methacrylates.
5. Decreased modulus of elasticity and cohesive strength of carious dentin.^15^

## CONCLUSION

Treating children often is challenged by the esthetic restoration of primary teeth that are discolored, malformed or have multiple surface carious or traumatic destructions. An effective bond to the primary dentin and enamel would reduce the failures and preserve the tooth structure by allowing more conservative cavity preparations. The present study concluded that the nanocomposite had higher shear bond strength both in carious and sound dentin compared to the conventional composites. However, the clinical procedure performed by the clinicians is the major consideration. Nonetheless, clinical implications of this research are significant.
